# Allelic Variation in *GmPAP14* Alters Gene Expression to Affect Acid Phosphatase Activity in Soybean

**DOI:** 10.3390/ijms24065398

**Published:** 2023-03-11

**Authors:** Youbin Kong, Yuan Liu, Wenlong Li, Hui Du, Xihuan Li, Caiying Zhang

**Affiliations:** 1 North China Key Laboratory for Crop Germplasm Resources of Education Ministry, Hebei Agricultural University, Baoding 071000, China; 2 State Key Laboratory of North China Crop Improvement and Regulation, Hebei Agricultural University, Baoding 071000, China

**Keywords:** purple acid phosphatase, allelic gene, transcriptional level, APase activity

## Abstract

Improvement in acid phosphatase (APase) activity is considered as an important approach to enhance phosphorus (P) utilization in crops. Here, *GmPAP14* was significantly induced by low P (LP), and its transcription level in ZH15 (P efficient soybean) was higher than in NMH (P inefficient soybean) under LP conditions. Further analyses demonstrated that there were several variations in gDNA (*G-GmPAP14Z* and *G-GmPAP14N*) and the promoters (*P-GmPAP14Z* and *P-GmPAP14N*) of *GmPAP14*, which might bring about differential transcriptional levels of *GmPAP14* in ZH15 and NMH. Histochemical staining measurements revealed that a stronger GUS signal was present in transgenic Arabidopsis with *P-GmPAP14Z* under LP and normal P (NP) conditions compared with the *P-GmPAP14N* plant. Functional research demonstrated that transgenic Arabidopsis with *G-GmPAP14Z* had a higher level of *GmPAP14* expression than the *G-GmPAP14N* plant. Meanwhile, higher APase activity was also observed in the *G-GmPAP14Z* plant, which led to increases in shoot weight and P content. Additionally, validation of variation in 68 soybean accessions showed that varieties with Del36 displayed higher APase activities than the del36 plant. Thus, these results uncovered that allelic variation in *GmPAP14* predominantly altered gene expression to influence APase activity, which provided a possible direction for research of this gene in plants.

## 1. Introduction

Phosphorus (P) is an essential macronutrient as it is a constituent of key macromolecules such as nucleic acids and phospholipids. It also plays a key role in the regulation of many enzymatic reactions, signal transduction processes and metabolic pathways. Low P seriously influences plant growth and development [1]. In fact, P is abundant in the soil, but a large proportion of P is usually present in the form of organic P (P_o_) or is bound with metal ions (iron and aluminum), and is thus unavailable for plant utilization [2,3]. To maintain or acquire high crop yields, plenty of phosphate (P_i_) fertilizers are commonly applied in agricultural production [4]. However, most phosphate fertilizers are not fully assimilated by plants and are immobilized with cations in the soil, which not only increases the economic burden of farmers but also causes soil degradation and water eutrophication [5]. Therefore, improving the efficiency of P acquisition and utilization is necessary for the green and sustainable development of agriculture [6].

During evolution, to deal with a low phosphorus environment, plants have developed a series of adaptive mechanisms to improve efficiency of P utilization, including modification of root architecture, improvement in acid phosphatase (APase) activity, remodeling of membrane lipids, etc. [7,8]. Of these various strategies, APase is considered to hydrolyze phosphoric acid monoesters and diesters or anhydrides to release P_i_, which is used as an essential parameter for choosing P efficient varieties [9]. These enzymes are generally divided into two groups, non-specific and specific, based on their particular catalyzing substrate.

Purple acid phosphatase (PAP) is a non-specific APase and contains a binuclear metal ion center. Moreover, PAPs have five conserved motifs (**D**XG/G**D**XX**Y**/G**N**H(D/E)/VXX**H**/G**H**X**H** (bold letters represent invariant residues)), which can coordinate the binuclear metal center to hydrolyze a range of organic phosphate (Po) [10,11,12]. In plants, many members of the PAP gene family have been identified, such as 29 PAPs in *Arabidopsis* [13], 26 PAPs in rice [14], 35 PAPs in soybean [15] and 33 PAPs in maize [16]. In addition, it has been verified that several of them participate in Po utilization. In *Arabidopsis*, *AtPAP10* [17] and *AtPAP26* [18,19] are two important PAP genes, whose ability to utilize Po have been well demonstrated. The AtPAP10 protein has phosphatase activity against a variety of substrates. Expression of *AtPAP10* is specifically induced by P_i_ limitation at both the transcriptional and post-transcriptional levels. Functional analyses of overexpressing lines indicated that *AtPAP10* plays an important role in plant tolerance to P_i_ limitation [17]. *Atpap26* mutant plants grew much smaller, had lower shoot and root APase activities and had reductions in free and total P_i_ concentration compared to the wild-type (WT) plants under a P starvation condition [19,20]. In rice, over-expression of *OsPAP10c* significantly enhanced APase activities in leaves, roots, root surface and culture media, and transgenic plants displayed higher tiller numbers under low P conditions [2]. Compared with WT plants, P_i_ concentrations in *OsPAP26*-overexpressing plants increased in the non-senescing leaves and decreased in the senescing leaves. *OsPAP26*-overexpressing plants exhibited a better growth when plants were grown in P_i_-depleted conditions [21].

Soybean is an important crop that provides a sustainable source of protein and oil worldwide. Some *GmPAPs* have been demonstrated to be involved in P_o_ decomposition and utilization. For instance, *GmPAP21* was induced by P_i_ limitation in nodules, roots and old leaves, and over-expression of *GmPAP21* significantly enhanced both APase activity and growth performance of hairy roots under P starvation conditions [22]. Another study pointed out that *GmPAP33* located at the plasma membrane participated in arbuscule degeneration during arbuscular mycorrhizal (AM) symbiosis *via* involvement in phospholipid hydrolysis [23]. *GmPAP7a* and *GmPAP7b*, the recently documented PAP genes, were upregulated by P_i_ starvation. Over-expression of *GmPAP7a* and *GmPAP7b* significantly improved root-associated APase activities and thus facilitated extracellular ATP utilization in soybean hairy roots [10]. In our previous studies, *GmPAP14* has been demonstrated to be an important gene in response to P_o_, and it predominantly participates in utilizing external P_o_ to enhance plant growth and development [24]. In this paper, we found that *GmPAP14* had several allelic variations and investigated the relationship between allelic variation and APase activity in soybean.

## 2. Results

### 2.1. GmPAP14 Was Significantly Induced in Roots of ZH15 under Low P Conditions

**Figure 1:**
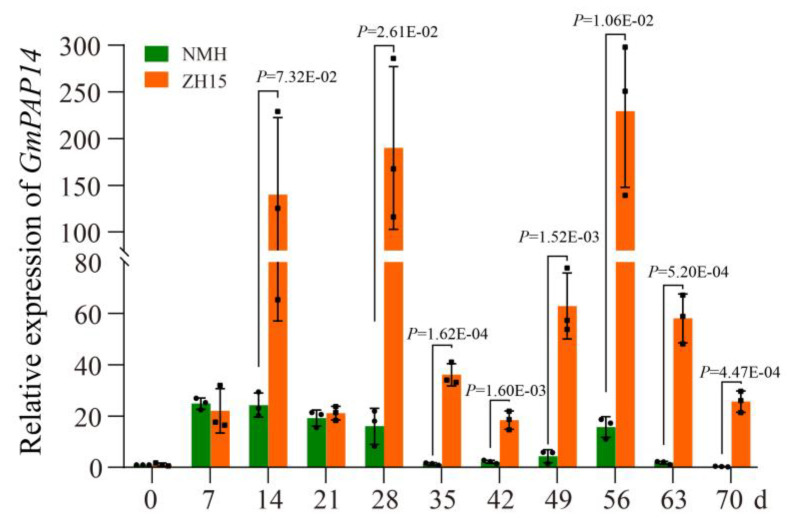
Analysis of *GmPAP14* expression in roots of zhonghuang (ZH15, P efficient soybean) and niumaohuang (NMH, P inefficient soybean). Seven-day-old seedlings were treated with normal P (NP, 1 mmol/L KH_2_PO_4_) and low P (LP, 1 mmol/L phytate). Seedlings treated with NP were used as a control. The roots were sampled after 7, 14, 21, 28, 35, 42, 49, 56, 63 and 70 days and were used for temporal expression analysis. The relative expression was calculated using the 2^−ΔΔCt^ method, and all data represent the means ± SD, *n* = 3. Error bars represent the SD. A *t*-test was used to identify the differences between the data.

In this paper, we first analyzed the temporal expression of *GmPAP14* in roots of zhonghuang15 (ZH15, P efficient soybean) and niumaohuang (NMH, P inefficient soybean) under low P (LP) conditions. The results of quantitative PCR (qPCR) revealed that the expressional pattern of *GmPAP14* contained observable differences between the two varieties (Figure 1). In ZH15, *GmPAP14* was strongly induced after 7 days (d) post LP conditions (DPP), and its expression was maintained at a high level from 14–70 DPP, with the peak occurring at 56 DPP. In contrast, expression of *GmPAP14* in NMH was only higher at 7–28 and 56 DPP, and was relatively consistent at other time points. However, a comparative analysis showed that ZH15 exhibited significantly higher expression levels than NMH at 14 DPP and 28 to 70 DPP, implying that the regulation of *GmPAP14* at the transcriptional level may be diverse.

### 2.2. Variation in the GmPAP14 Promoter Affected Gene Expression

To explore what caused the different expressional levels of *GmPAP14*, we first cloned *GmPAP14* promoter sequences in ZH15 and NMH. The results displayed that the length of *GmPAP14* promoter was 1635 bp in ZH15 (*P*-*GmPAP14Z*) and 1643 bp in NMH (*P*-*GmPAP14N*). Comparative analyses of sequences showed that there were 1-bp, 2-bp and 7-bp insertions/deletions (InDels) and 11 SNPs between them (Supplementary Figure S1). These differences might bring about variations in promoter regulatory elements. Subsequently, the regulatory elements of promoters were predicted by the PLACE [25] and PlantCAR [26] databases. We found that two stress response regulatory elements (RAV1BAT and ACGTTBOX) were especially present in *P*-*GmPAP14Z* but not in *P*-*GmPAP14N*. Moreover, there was a negative regulatory element (NRRBNEXTA) in *P*-*GmPAP14N* (Supplementary Table S2).

**Figure 2:**
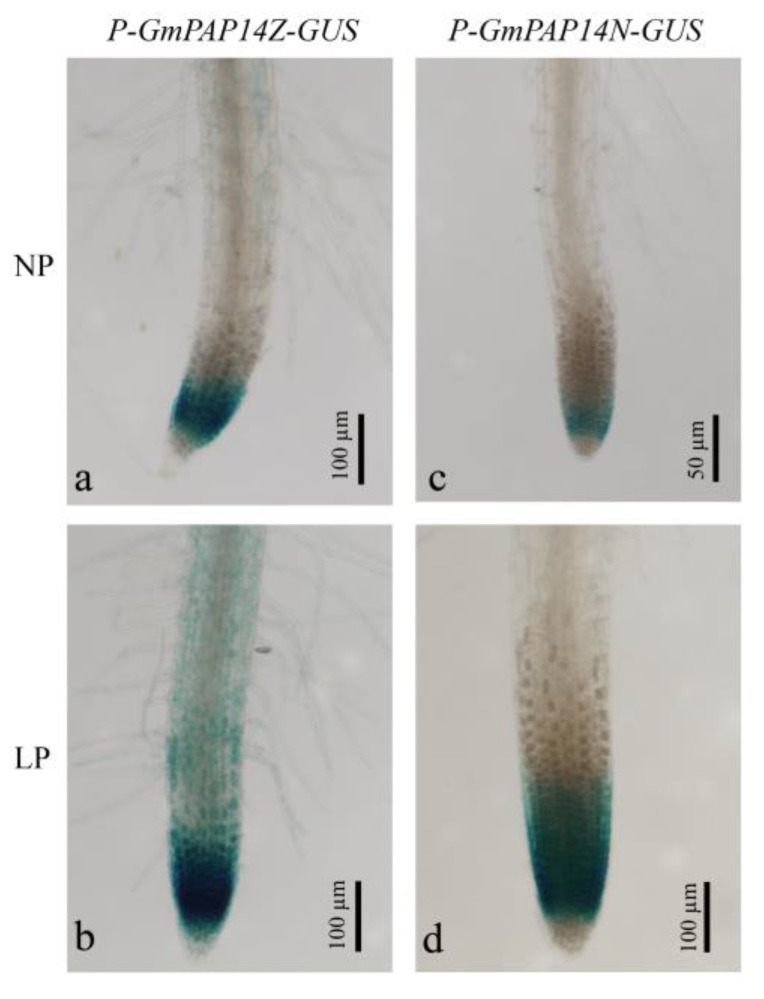
Variational analysis of the *GmPAP14* promoter in roots of transgenic *Arabidopsis*. The T_3_ transgenic plants with *P-GmPAP14Z-GUS* and *P-GmPAP14N-GUS* were grown on agar under normal P (NP, 1 mmol/L KH_2_PO_4_) and low P (LP, 1 mmol/L phytate) conditions. Then, the roots were harvested for GUS staining after 21 d.

To confirm the relationship between variation and gene expression, we constructed *P-GmPAP14Z-GUS* and *P-GmPAP14N-GUS* and transferred them into *Arabidopsis*. Then, we measured the GUS signals in the roots of transgenic *Arabidopsis* at 21 d after NP and LP stress (Figure 2). Stronger GUS signals were observed in transgenic roots with both *P-GmPAP14Z-GUS* and *P-GmPAP14N-GUS* under LP conditions, compared with those under NP conditions. However, both under LP and NP conditions, the GUS signals in roots of transgenic plants with *P-GmPAP14Z-GUS* were stronger than that in roots of transgenic plants with *P-GmPAP14N-GUS*. These results suggested that variations in the *GmPAP14* promoter affected the gene expression in the roots of ZH15 and NMH.

### 2.3. GmPAP14 gDNA Sequences Were Variational between ZH15 and NMH

**Figure 3:**
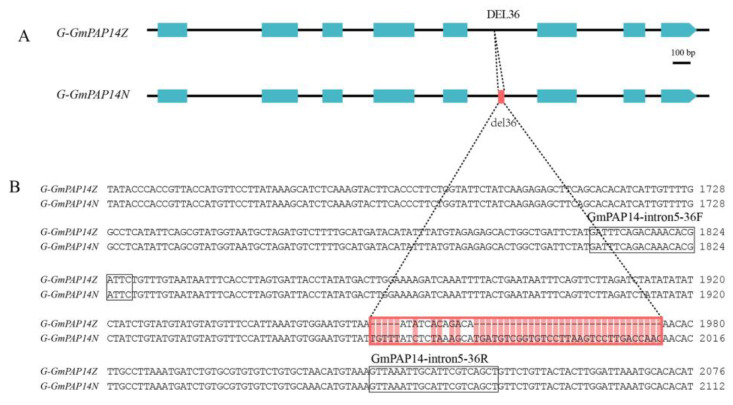
The structural analysis of *GmPAP14* gDNA in zhonghuang (ZH15) and niumaohuang (NMH). (A) The structure of *GmPAP14* gDNA. *G-GmPAP14Z* represents *GmPAP14* gDNA in ZH15 and *G-GmPAP14N* represents *GmPAP14* gDNA in NMH. The blue box represents exons and the black polylines represent introns. The red box represents differences in the two sequences. The marker GmPAP14-intron5-36F/R was designed according to the 36-bp variation, and was used for genotyping *GmPAP14* in soybean varieties.

**Figure 4:**
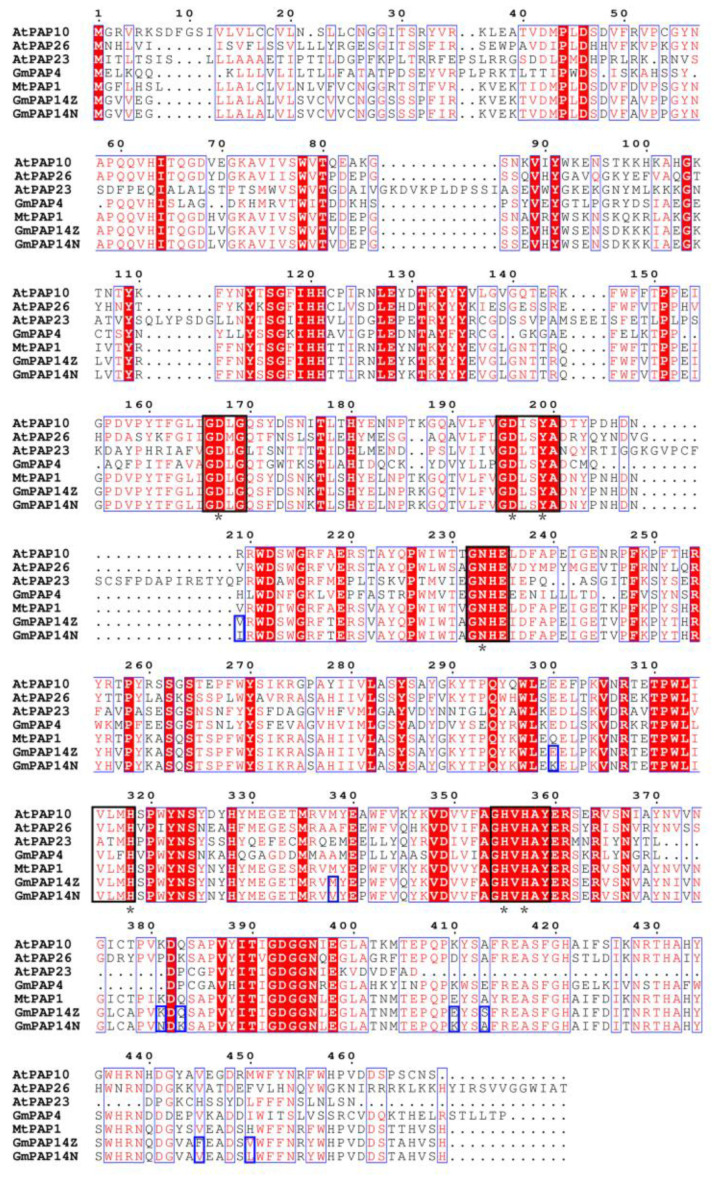
Amino acid sequence alignment of GmPAP14 and other PAPs. GmPAP14Z represents GmPAP14 protein in ZH15 and GmPAP14N represents GmPAP14 protein in NMH. Conserved motifs are indicated by black boxes. Conserved activity sites are indicated by asterisks. The variants of amino acid residues between GmPAP14Z and GmPAP14N are indicated by dark blue boxes. Protein alignment was performed by MEGA X and modified by ESPript3.0.

Furthermore, we cloned and found that the length of *GmPAP14* gDNA was 3040 bp in ZH15 (*G-GmPAP14Z*) and was 3076 bp in NMH (*G-GmPAP14N*). The results also showed that a 36-bp deletion (DEL36) was in the fifth intron of *G-GmPAP14Z* and 38 SNPs were distributed in them (Figure 3 and Supplementary Figure S2). However, a sequencing analysis showed that the lengths of *GmPAP14* cDNA were both 1395 bp in ZH15 and NMH (Supplementary Figure S3), which indicated that these variations had no effect on the splice site of *GmPAP14*. In addition, we compared protein sequences and found that nine amino acid residues of GmPAP14 were different between two varieties, separately located at 204 (V/I), 295 (E/K), 333 (M/V), 376 (K/N), 378 (Q/K), 405 (E/K), 408 (S/A), 440 (F/V) and 445 (V/L). Nevertheless, through aligning GmPAP14 with other PAPs, we discovered that their conserved motifs and enzymic sites were not modified (Figure 4), implying that these variations might mainly affect gene function at the transcriptional level.

### 2.4. G-GmPAP14Z Exhibited Higher Levels of GmPAP14 Expression and Significantly Improved Growth of Arabidopsis under Low P Conditions

**Figure 5:**
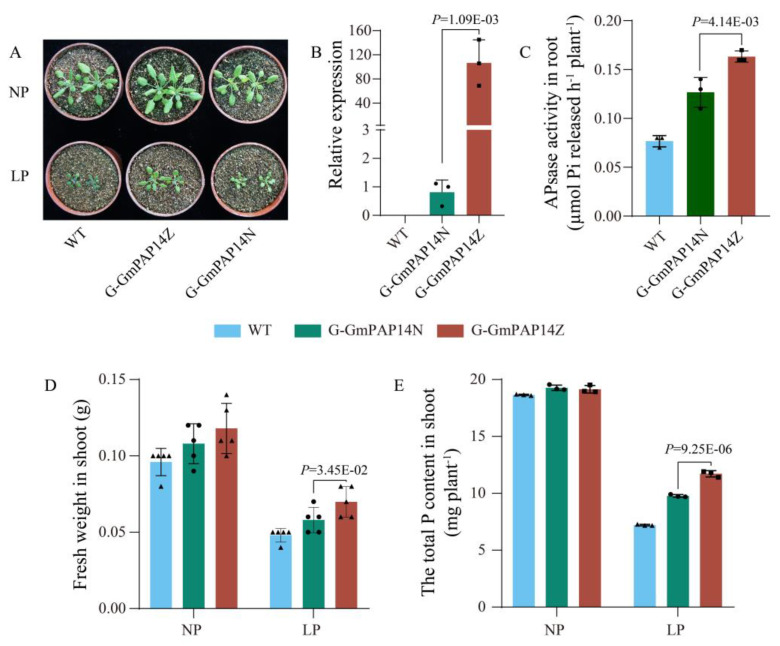
Variational analysis of the *GmPAP14* gDNA in transgenic *Arabidopsis*. (**A**) Appearance of 30-day-old seedlings after treatment with normal P (NP, 1 mmol/L KH_2_PO_4_) and low P (LP, 1 mmol/L phytate). (**B**) Relative expressional analysis of *GmPAP14* in transgenic plants with *G*-*GmPAP14Z* and *G*-*GmPAP14N*. All data represent the means ± SD, *n* = 3. Error bars represent the SDs. (**C**) Secreted APase activities in the roots of transgenic and wild-type plants. APase activity was expressed as ρ-NP released per hour per plant. All data represent the means ± SD, *n* = 3. Error bars represent the SDs. (**D**) Fresh weights of shoots of transgenic and wild-type plants under LP and NP conditions. All data represent the means ± SD, *n* = 5. Error bars represent the SDs. (**E**) Measurements of P content in shoots. After 30 days of growth, shoots were harvested separately for P content measurements. WT, wild-type plant; G-GmPAP14Z, transgenic plants with *G-GmPAP14Z*; G-GmPAP14N, transgenic plants with *G-GmPAP14N*. The data are the means ± SD, *n* = 3. Error bars represent the SD. One-way ANOVA was used to identify the differences between the data.

To investigate whether variations in *GmPAP14* gDNA affected gene transcription and function, we introduced *G-GmPAP14Z* and *G-GmPAP14N* into *Arabidopsis*. When P was in short supply, the transgenic plant with *G-GmPAP14Z* grew much better than the *G-GmPAP14N* plant (Figure 5A). We further investigated the expression of *GmPAP14* in the transgenic and wild-type plants by qPCR, and found that the level of *GmPAP14* expression in the *G-GmPAP14Z* plant was much higher than that in the *G-GmPAP14N* plant (Figure 5B). These results demonstrated that variations in *GmPAP14* gDNA also affected gene expression. Subsequently, we assessed the APase activities of transgenic and wild-type plants. The *G-GmPAP14Z* plant displayed higher APase activity (Figure 5C) compared with the *G-GmPAP14N* plant. In addition, under LP conditions, the shoot weight (Figure 5D) and P content (Figure 5E) of the *G-GmPAP14Z* plants were also significantly increased by 18.0% and 20.6%, respectively, compared with the *G-GmPAP14N* plant. These results indicated that *G-GmPAP14Z* should be superior to *G-GmPAP14N* in P utilization.

### 2.5. Allelic Variation in GmPAP14 was Closely Related to APase Activity in Soybean

**Figure 6:**
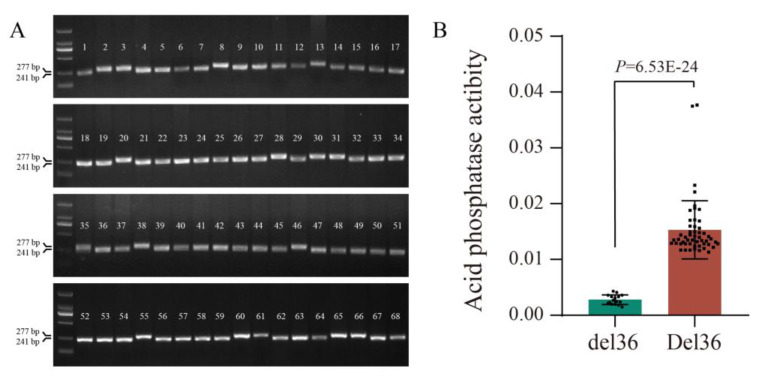
Genotypic and phenotypic analysis of 68 soybean varieties. (**A**) Amplification of *GmPAP14* allelic variants in 68 soybean varieties using the marker GmPAP14-intron5-36F/R. (**B**) Comparison of APase activity between two different genotypes. Del36 represents the variant in the fifth intron of *G-GmPAP14Z* and del36 represents the variant in the fifth intron of *G-GmPAP14N*. All values represent the means ± SD, *n* = 3. Error bars represent the SD. A *t*-test was used to identify the differences between the data.

Based on above results, we considered that the allelic variation in *GmPAP14* was closely related to APase activity in soybean. To further verify this, we designed a specific marker (GmPAP14-intron5-36F/R) according to Del36 in the fifth intron (Figure 3), and assayed it in 68 soybean varieties. The results revealed that 52 varieties with a 241-bp amplicon displayed significantly higher APase activities; in comparison, 16 of these varieties with a 277-bp amplicon had low APase activities (Figure 6), explaining that this allelic variation in *GmPAP14* played an important role in APase activity. This provided a potential site for screening soybean varieties with high APase activity.

## 3. Discussion

Purple acid phosphatase (PAP) belongs to the metallophosphatase superfamily proteins, carrying a metallophos domain and a bimetallic reaction center at active sites [27]. It has been functionally characterized in P_o_ utilization in several plants, including *Arabidopsis* [12], rice [21], soybean [10,28] and barley [29]. The amino acid sequence analyses of mammal and plant PAPs have revealed that the presence of five conserved domains/motifs (**D**XG/G**D**XX**Y**/G**N**H(D/E)/VXX**H**/G**H**X**H** (bold letters represent invariant residues)) contributes to the enzyme activity [10,11]. Similar conclusions were also found in studies of the fragrance in vegetable soybean and rice. Conserved protein sequences are believed to be essential for the functional activity of betaine aldehyde dehydrogenase [30]. In this paper, we found that there were some allelic variations between *G-GmPAP14Z* and *G-GmPAP14N*, especially a 36-bp InDel in their fifth introns (Figure 3 and Supplementary Figure S2). Additionally, these variations caused differences in nine amino acid residues between GmPAP14Z and GmPAP14N, whereas the alignments of protein sequences with other PAPs showed that their conserved motifs were not changed (Figure 3), suggesting that these changes had little effect on APase activity.

Many studies have demonstrated that *cis*-regulatory elements are important mediators of PAPs in response to low P stress [2,21]. In the current study, we cloned promoter sequences of *GmPAP14* in ZH15 (*P-GmPAP14Z*) and NMH (*P-GmPAP14N*). Bioinformatic analyses showed that there were some allelic variations between them, bringing about two *cis*-elements, RAV1BAT (RAV, AP2 domain transcription factor binding site) [31] and ACGTTBOX (bZIP transcription factor binding site) [32], only in *P-GmPAP14Z*. Many studies have demonstrated that AP2 and bZIP transcription factors are involved in a variety of abiotic stress responses in plants [33]. Additionally, a quantitative analysis showed that *GmPAP14* was significantly induced in ZH15 when compared to NMH from 28 d to 70 d under LP conditions. Meanwhile, histochemical staining showed that the GUS signal in the roots of transgenic plants with *P-GmPAP14Z-GUS* was more intense than that in the roots of transgenic plant with *P-GmPAP14N-GUS* under LP and NP conditions (Figure 2). These results suggested that the two special *cis*-elements might account for the higher level of *GmPAP14* expression in ZH15 under LP condition.

Since the discovery of introns, there have been considerable efforts to understand their functions and evolution. A growing number of studies have demonstrated that introns are involve in gene regulation *via* alternative splicing [34]. In an earlier study, researchers have found that a 191-bp insertion in the intron affected the expression levels of the polyphenol oxidase gene, resulting in a lower PPO activity in wheat [35]. Recent research has also reported that THP9 encoded an asparagine synthetase 4 enzyme which was highly expressed in teosinte, but not in the B73 inbred, in which a deletion in the tenth intron of THP9-B73 caused incorrect splicing of THP9-B73 transcripts [36]. In this paper, we also induced a 36-bp deletion (Del36) in the fifth intron of *G-GmPAP14Z*. Subsequent experiments showed that the transgenic plant with *G-GmPAP14Z* possessed a much higher transcriptional level of *GmPAP14* (Figure 5B) and APase activity (Figure 5C). Additionally, significant increases in the weights of the shoots (Figure 5D) and in the P contents of the shoots (Figure 5E) were observed in transgenic plants overexpressing *G-GmPAP14Z* under LP conditions. Therefore, we considered that Del36 was closely associated with a much higher transcriptional level of *GmPAP14* in ZH15 under LP condition.

Allelic variation in gene helps us not only to comprehend the mechanisms underlying phenotypic variation, but also to screen certain varieties via markers developed based on variation [37]. In rice, several allele-specific markers of *OsPSTOL1* were designed for molecular breeding to improve their low P tolerance [38]. In this paper, a specific marker was designed to distinguish APase activities in soybean varieties. Fortunately, varieties with Del36 displayed higher APase activities; in contrast, varieties with del36 displayed lower APase activities (Figure 6), providing a potential tool for selecting higher APase activity soybeans.

In summary, we identified allelic variations in *GmPAP14* between P efficient and P inefficient soybean varieties. Our data indicated that these allelic variations predominantly determined gene transcription levels to affect acid phosphatase activity of the roots in soybean. Additionally, a specific marker designed based on the Del36 in the fifth intron is as a potential molecular tool for screening high APase activity soybean varieties.

## 4. Materials and Methods

### 4.1. Plant Materials and Growth Conditions

The soybeans zhonghuang15 (ZH15, P efficient soybean) and niumaohuang (NMH, P inefficient soybean) were used for gene cloning. These two genotypes had morphological and physiological differences under P starvation conditions, such as the relative values of shoot dry weight (0.85, 0.52) and root dry weight (1.06, 0.80) [28,39]. For allelic variation analysis, 68 soybean varieties (Supplementary Table S3) were used. The normal P (NP) and low P (LP) conditions used in this paper were carried out using a modified Hoagland solution with 1 mmol/L KH_2_PO_4_ and 1 mmol/L phytate, respectively.

### 4.2. Quantitative RT-PCR

The seeds of ZH15 and NMH were placed in pots with vermiculite in a greenhouse (12 h light, 28 °C and 12 h dark, 24 °C, relative humidity 60%). After 7 days (d) of growth (0 d was used as a control), the seedlings were separately treated with a modified Hoagland solution with 1 mmol/L KH_2_PO_4_ (NP, as controlled) and 1 mmol/L phytate (LP) once a week. Then, the roots were harvested for temporal gene expression profiling after 0, 7, 14, 21, 28, 35, 42, 49, 56 and 70 d. Total RNA was extracted using an RNAprep Pure Plant Kit (Tiangen, Beijing, China). Subsequently, the synthesis of cDNA was performed with a PrimeScriptTM Reagent kit (Takara Bio, Dalian, China). Quantitative RT-PCR (qPCR) was carried out with the EvaGreen^®^ qPCR Master Mix (US Everbright^®^ Inc., Suzhou, China) on a CFX96 Real-Time PCR Detection System (Bio-Rad, Hercules, USA). The primers of *GmPAP14* and the housekeeping gene *GmActin11* are listed in Supplementary Table S1. Relative expression was calculated using the 2^−ΔΔCt^ method [40]. Three replicates were performed for all PCR samples.

### 4.3. Cloning of GmPAP14 cDNA, Genomic DNA and Promoter Sequences in Soybean

To obtain the cDNA sequence of *GmPAP14*, total RNA was extracted from the roots of ZH15 and NMH using an RNAprep Pure Plant Kit (Tiangen). Then, the first-strand cDNA was synthesized with a PrimeScript^TM^ Reagent kit and the gDNA Eraser (Takara). Finally, the primers F1/R1 (Supplementary Table S1) were used for amplification of the full-length cDNA of *GmPAP14* in ZH15 (*GmPAP14Z*) and NMH (*GmPAP14N*). To obtain the genomic DNA (gDNA) sequence of *GmPAP14*, total DNA was extracted from the roots of ZH15 and NMH with the CTAB method. Subsequently, the gDNA sequences of *GmPAP14* were amplified in ZH15 (*G-GmPAP14Z*) and NMH (*G-GmPAP14N*) with the primers F1/R1 (Supplementary Table S1), respectively. For cloning of the *GmPAP14* promoter, sequences of the *GmPAP14* promoter were amplified in ZH15 (*P-GmPAP14Z*) and NMH (*P-GmPAP14N*) using the primers F2/R2 (Supplementary Table S1).

### 4.4. Vector Construction and Plant Transformation

To construct the *GmPAP14* gDNA vector, *G-GmPAP14Z* and *G-GmPAP14N* were separately inserted into a pBI121 vector that had been digested with *Xba* I and *Sac* I. To construct the promoter vector, the amplified fragments of *P-GmPAP14Z* and *P-GmPAP14N* were digested with *Hind* III and *BamH* I and cloned into the pCamG vector. The above constructed plasmids were imported into *Agrobacterium tumefaciens* GV3101 using a freeze–thaw procedure and transgenic *Arabidopsis* were generated via *Agrobacterium*-mediated floral dip.

### 4.5. Histochemical GUS Staining

To estimate allelic variations of promoters affecting *GmPAP14* expression, T_3_ transgenic plants with *P-GmPAP14Z-GUS* and *P-GmPAP14N-GUS* were grown on agar under NP and LP conditions. Then, the roots were harvested for GUS staining after 21 d. The samples were incubated for 5 h in GUS staining buffer (2 mmol/L 5-bromo-4-chloro-3-indolyl-bglucuronic acid in 50 mmol/L sodium P_i_ buffer, pH 7.2) containing 0.1% Triton X-100, 2 mmol/L K_4_Fe(CN)_6_, 2 mmol/L K_3_Fe(CN)_6_ and 10 mmol/L EDTA·Na_2_. Then, the stained samples were observed and imaged using a BX51 microscope (Olympus, Tokyo, Japan) [24].

### 4.6. Measurement of APase Activity in Transgenic Arabidopsis

APase activity was analyzed as previously described in [24]; 15-day-old seedlings grown under LP conditions were transferred to 2 mL Eppendorf tubes containing 1.5 mL of a liquid medium supplemented with 1 mmol/L ρ-NPP (ρ-NPP, Sigma, Darmstadt, Germany). After being maintained for 1 day at 24 °C, 0.5 mL of 0.5 mmol/L NaOH was added to terminate the reaction. Absorbance was measured at 410 nm. APase activity was expressed as ρ-NP released per hour per plant. All experiments were repeated three times, with three plants per replication.

### 4.7. Measurement of P Content in Transgenic Arabidopsis

The wild-type (as controlled) and transgenic *Arabidopsis* with *G-GmPAP14Z* and *G-GmPAP14N* were planted and treated under NP and LP conditions in a greenhouse (12 h light, 28 °C and 12 h dark, 24 °C, relative humidity: 60%). After a 30-d treatment, the sampled fresh shoots were dried at 80 °C for 24 h and weighed. Then, the samples were flamed to ashes, which were subsequently incubated in 100 μL of 30% HCl and 10% HNO_3_. Next, 20 μL of the dissolved sample was mixed with 500 μL of P reaction buffer (5% ammonium molybdate solution and 10% ascorbic acid; 6:1, *v*:*v*), and then incubated at 37 °C for 1 h. Finally, P content was determined at 820 nm with a spectrophotometer [2]. All experiments were repeated three times, with three plants per replication.

### 4.8. Variation Analysis of GmPAP14 in Natural Soybean Populations

Based on the 36-bp allelic variation in the fifth intron of *GmAP14*, we designed the marker GmPAP14-intron5-36 (F: 5′-GATTTCAGACAAACACGATTC-3′; R: 5′-AGCTGACGAATGCAATTTAAC-3′) for genotyping varieties. Natural soybean populations were planted in greenhouse under LP conditions. After 30 d, APase activities in roots were measured for genotyping with developed markers (GmPAP14-intron5-36 F/R). The method of measurement of APase activity was as follows: the total protein was first extracted with a Plant Protein Extraction Kit (CWBIO, Taizhou, China) from root samples of 30-day-old seedlings. Subsequently, 20 μL of total protein was incubated with 1 mmol/L ρ-nitrophenol phosphate (ρ-NPP, Sigma) at 37 °C for 30 min in 1480 μL of NaOAc buffer (200 μmol L^−1^; pH 5.0). Afterwards, the reaction was terminated by adding 500 μL of NaOH (0.5 mol/L) for a total volume of 2.0 mL. Finally, the reaction product, ρ-nitrophenol (ρ-NP), was measured spectrophotometrically at 410 nm [24]. All experiments were repeated three times, with three plants per replication.

### 4.9. Data Analysis

All data were analyzed using SPSS 17.0 software (IBM, Armonk, NY, USA). One-way ANOVA and a *t*-test were used to identify the differences between the observations. The pictures were drawn in GraphPad Prism 8.0 (GraphPad Software, San Diego, CA, USA).

## Data Availability

The data supporting the findings of this study are available within the article and its Supplementary Materials.

## Supplementary Materials

The supporting information can be downloaded at: https://www.mdpi.com/article/10.3390/ijms24065398/s1.
