# Supplementary material for: Allelic Variation in GmPAP14 Alters Gene Expression to Affect Acid Phosphatase Activity in Soybean
Source: Int J Mol Sci. 2023 Mar 11;24(6):5398. doi: 10.3390/ijms24065398 (PMC10049298; doi:10.3390/ijms24065398)
Supplement: Supplementary file 1 [file ijms-24-05398-s001.zip › Supplementary Figures S1-S3.pdf]

```
P-GmPAP14Z  CTGAGGAAAGACTACAGTATACTCTCTCTTTTTTGTCGTAAATTATAGTATAGTACTCTTTTCAATTAATATGTACTCTA 80
P-GmPAP14N  CTGAGGAAAGACTACAGTATACTCTCTCTTTTTTGTCGTAAATTATAGTATAGTACTCTTTTCAATTAATATGTACTCTA 80

P-GmPAP14Z  ATAAATATTGTTGATGCGTAGTCGATGGTTCACAACTAGTATCAAATAATTAGTAGTTGCGACTGATGGTCGATAACCAA 160
P-GmPAP14N  ATAAATATTGTTGATGCGTAGTCGATGGTTGACAACTAGTATCAAATAATTAGTAGTTGCGACTGATGGTCGATAACCAA 160

P-GmPAP14Z  CATCTCAATTGATATCCGAGACTTAATAGTTGAGAATAGTGATGCCCGATGGTCAACCTTAAATTAGTGGGATCAAGTTA 240
P-GmPAP14N  CATCTCAATTGATATCCGAGACTTAATAGTTGAGAATAGTGATGCCTGATGGTCAACCTTAAATTAGTGGGATCAAGTTA 240

P-GmPAP14Z  AGATCCATTAAGTTTAAATTGATAATTCAAGGGACCAAAAATCATATGAACTCATGAGACATAAGTTAGAACAAAACAAC 320
P-GmPAP14N  AGATCCATTAAGTTTAAATTGATAATTCAAGGGACCAAAAATCATATGAACTCATGAGACATAAGTTAGAAGAAAACAAC 320

P-GmPAP14Z  CGATGGTAGAGCCGAGCGACATTTCGGTTATGTTGGGCAAAAGTATCTATAAAGTTATTTGAAAAATGTAAGTAATAAAC 400
P-GmPAP14N  CGATGGTAGAGCCGAGCGACATTTCGGTTATGTTGGGCAAAAGTATCTATAAAGTTATTTGAAAAATGTAAGTAATAAAC 400

P-GmPAP14Z  GATTGCAATGTAAGTAGATTTTAAGGGACAAATCTTTTTTCTGTAAAAAGAGGGATGAATCTT--ATCAAATTATTTAAT 478
P-GmPAP14N  GATTGCAATGTAAGTAGATTTTAAGGGACAAATCTTTTTTCTGTAAAAAGAGGGATGAATCTTTTATCAAATTATTTAAT 480

P-GmPAP14Z  GCCTTACCATCAACTTCTCAATTTAATGCCTTGCCATCGATTTCTTAAATTAATAATTGGCATGCATTTTTGTTTCCAAA 558
P-GmPAP14N  GCCTTACCATCAACTTCTCAATTTAATGCCTTGCCATCGATTTCTTAAATTAATAATTGGCATGCATTTTTGTTTCCAAA 560

P-GmPAP14Z  AAAATTCACAACATTTATATTAAAAAAAAAGTATTCGCATGTCATTATTAAATTTGAGAATTTAAAGATAAGGTGCACCC 638
P-GmPAP14N  AAAATTCACAACATTTATATTAAAAAAAAAGTATTCGCATGTCATTATTAAATTTGAGAATTTAAAGATAAGGTGCACCC 640

P-GmPAP14Z  AACTTTATTAATCTCTCCTTTTATATGTTCTTAAGTTATAGTATTTAGAAAATATTAAATGTAACGATATTTTTTTCCAT 718
P-GmPAP14N  AACTTTATTAATCTCTCCTTTTATATGTTCTTAAGTTATATTATTTAGAAAATATTAAATGCAACGAT-TTTTTTTCCAT 719

P-GmPAP14Z  TGGGGTTAAAAACAATGCAAATATGTAGCCGATCTTATAAAATAATTATCATAGTGAAATAATTTTATTTAAGATGGATA 798
P-GmPAP14N  TGGGGTTAAAAATAATGCAAATATGTAGCCGATCTTATAAAATAATTATCGTAGTGAAATAATTTTATTTAAGATGGATA 799

P-GmPAP14Z  AGATTATTATAGGACCGATTAAAATAAAAGAATCCAATCCAC-------CTGTTGATTGATGGTTGATGATAAGAGAGAA 871
P-GmPAP14N  AGATTATTATAGGACCGATTAAAATAAAAGAATCCAATCCACTAATTAGCTGTTGATTGATGGTTGATGATAAGAGAGAA 879

P-GmPAP14Z  GTTAATTAAGAGTAAAACAAAGGGTATATCATATCATAACGTTTGTAACTTTTATGAAATAACACTAGCCTATAATTTCA 951
P-GmPAP14N  GTTAATTAAGAGTAAAACAAAGGGTATATCAAATCATAACATTTGTAACTTTTATGAAATAACACTAGCCTATAATTTCA 959

P-GmPAP14Z  TTTGATTATGACCTTTTCTTTTTCAAAATGACATAGAAAAATGTAATCAATGATCACAATAAAAAAGATTCCAATTTGTT 1031
P-GmPAP14N  TTTGACTATGACCTTTTCTTTTTCAAAATGACATAGAAAAATGTAATCAATGATCACAATAAAAAAGATTCCAATTTGTT 1039

P-GmPAP14Z  TGACGCCATATATAGAAAAGTGTGTCTCCAACTCTTAACTGGGGCTATCCGGCCCATTCGGACAACTTGTATGGTCTGAA 1111
P-GmPAP14N  TGACGCCATATATAGAAAAGTGTGTCTCCAACTCTTAACTGGGGCTATCCGGCCCATTCGGGCAACTTGTATGGTCTGAA 1119

P-GmPAP14Z  CTGAACGCCACTCCTCATATGCTGCAATTCCAATTCGATTCAGACCTGTGCATTCAAAGTCAACTTTGTAATTTGTAACA 1191
P-GmPAP14N  CTGAACGCCACTCCTCATATGCTGCAATTCCAATTCGATTCAGACCTGTGCATTCAAAGTCAACTTTGTAATTTGTAACA 1199

P-GmPAP14Z  AAAATCTTGGCTGAACAACTTCTTTTTCCTATGAGATGGAAGAAAAAATAAATAAGTGAGAGAGATACATGATGTGACGT 1271
P-GmPAP14N  AAAATCTTGGCTGAACAACTTCTTTTTCCTATGAGATGGAAGAAAAAATAAATAAGTGAGAGAGATACATGATGTGACGT 1279

P-GmPAP14Z  GAATCAATATTATATTATAGTTTATACTTTATCATTATTTTTATCAGTTCACCGCTTCAACTTAACTGAAGCCATTCATT 1351
P-GmPAP14N  GAATCAATATTATATTATAGTTTATACTTTATCATTATTTTTATCAGTTCACCGCTTCAACTTAACTGAAGCCATTCATT 1359

P-GmPAP14Z  GGTTTGTTTCTCTTAGTCTTCGCGGTTAACGTAAACTGAACCGTGTTTTTGTCTTTCGAAGAAAACGATGGTAAATGTTG 1431
P-GmPAP14N  GGTTTGTTTCTCTTAGTCTTCGCGGTTAACGTAAACTGAACCGTGTTTTTGTCTTTCGAAGAAAACGATGGTAAATGTTG 1439

P-GmPAP14Z  CTTGCTTCACGGACCCTCTGTCTTGTTGACCTCTAATGTGAATATTCCTCATCATATGCCATTCCATTCATTTAACAGTT 1511
P-GmPAP14N  CTTGCTTCACGGACCCTCTGTCTTGTTGACCTCTAATGTGAATATTCCTCATCATATGCCATTCCATTCATTTAACAGTT 1519

P-GmPAP14Z  AATGCACCCTTATCAATTTACCTTATACCAGTACTTATCATTATAAAGATCAGAGAGTGCATTGGAATAATAAATACGCG 1591
P-GmPAP14N  AATGCACCCTTATCAATTTACCTTATACCAGTACTTATCATTATAAAGATCAGAGAGTGCATTGGAATAATAAATACGCG 1599

P-GmPAP14Z  AGTGTTGCGACCCTGGATCACTAGAATCACGGGTTTTGAGCAAA                                     1635
P-GmPAP14N  AGTGTTGCGACCCTGGATCACTAGAATCACGGGTTTTGAGCAAA                                     1643
```

Figure S1 : Sequence alignment of *P-GmPAP14Z* and *P-GmPAP14N*.

```
G-GmPAP14Z   ATGGGTGTTGTGGAGGGTCTCTTAGCATTGGCTTTGGTTCTGAGTGTTTGCGTGGTGTGCAATGGAGGCTCAAGCAGCCCCTTCATTAGGAAAGT 95
G-GmPAP14N   ATGGGTGTTGTGGAGGGTCTCTTAGCATTGGCTTTGGTTCTGAGTGTTTGCGTGGTGTGCAATGGAGGCTCAAGCAGCCCCTTCATTAGGAAAGT 95

G-GmPAP14Z   TGAGAAGACAGTGGATATGCCACTTGACAGCAATGTCTTTGCTGTTCCTCCTGGTTATAATGCTCCCCAGCAGGTATCTACCTACCCTTCTCTTC 190
G-GmPAP14N   TGAGAAGACAGTGGATATGCCACTTGACAGCGATGTCTTTGCTGTTCCTCCTGGTTATAATGCTCCCCAGCAGGTACCTACCTACCCTTCTCTTC 190

G-GmPAP14Z   TTCTTCTTTACTATCTTCATTTTTAATTCTGGTTTTCTGTTAGAGTAGAGAGGGAAGTGGTTAATGATGCTTTGCTGCCCAGTGAATCAAGTTCT 285
G-GmPAP14N   TTCTTCTTTACTATCTTCATTTTTAATTCTGGTTTTCTGTTAGAGTAGAGAGGGAAGTGGTTAATGATGCTTTGCTGCCCAGTGAATCAAGTTCT 285

G-GmPAP14Z   GTACTTGCCATTAACCAATGAGTGAAATTTAGATGCAGTTTTATGCTTGTGTGCCATTCTCCAATTCAAATTAGAGTTTCACTTTGATAATTTGA 380
G-GmPAP14N   GTACTTGCCATCAACCAATGAGTGAAATTTAGATGCAGTTTTATGCTTGTGTGCCATTCTCCAATTCAAATTAGAGTTTCACTTTGATAATTTGA 380

G-GmPAP14Z   GGTGATGTTTGATAGAAATCTTTATTATCATCATATTATCCAAGTGAAATTTCAGTTCTGATACCAGCATAGCACCAAAAACACCAATAGAGCTA 475
G-GmPAP14N   GATGATGTTTGATAGAAATCTTTATTATCATCATATTATCCAAGTGAAATTTCAGTTCTGATACCAGCATAGCACCAAAAACACCAATAGAGCTA 475

G-GmPAP14Z   CATCTATGCTTTGTGCTTAACCAATTCTCTTTCTAAATTCAACATTTTTACATTTTTGCACCCTTTGGGATTTTTTCTTTCTTTGATTTGAATCA 570
G-GmPAP14N   CATCTATGCTTTGTGCTTAACCAATTCTCTTTCTAGATTCAACATTTTTACATTTTTGCACCCTTTGGGATTTTTTCTTTCTTTGATTTGAATCA 570

G-GmPAP14Z   ATGGCTCCTATGCATTAAGGTTCATATAACGCAAGGTGACCTTGTGGGGAAAGCAGTGATCGTGTCATGGGTGACAGTGGATGAACCGGGGTCGA 665
G-GmPAP14N   ATGGCTCCTATGCATTAAGGTTCATATAACGCAAGGTGACCTTGTGGGGAAAGCAGTGATCGTGTCATGGGTGACAGTGGATGAACCGGGGTCGA 665

G-GmPAP14Z   GCGAAGTGCATTACTGGAGTGAGAACAGTGACAAGAAGAAGATTGCCGAAGGAAAACTTGTTACTTATAGGTTCTTCAATTACACATCTGGTTTT 760
G-GmPAP14N   GCGAAGTGCATTACTGGAGTGAGAACAGTGACAAGAAGAAGATTGCCGAAGGAAAACTTGTTACTTATAGGTTCTTCAATTACTCATCTGGGTTT 760

G-GmPAP14Z   ATTCATCACACCACCATCAGAAATTTGGAGGTAATTCTCTTTTCCATTACTTTGGCTTTATGCTGTTTATTGTCCAGATAGATGCCAGGCTAGAA 855
G-GmPAP14N   ATACACCACACCACCATCAGAAATTTGGAGGTAATTATCTTTTCCATTACTTTGGTTCTATGCTGCTTATTGTCCAGATAGATGCCAAGCTAGAA 855

G-GmPAP14Z   TCTTGTGAAAGAAAAGAAAAAGGGCATTTTGCTTTGTGAATCAATTGTTGATACGTGTGCTTCTTTTGGGGCAGTACAACACCAAATACTACTAT 950
G-GmPAP14N   GCTTGGGAAAGAAAATAAAAAGGGCATTTTGCTTTGTGTATCAATTGTTGATATGTGTGCTTCTTTTGGGGCAGTACAAAACCAAATACTACTAT 950

G-GmPAP14Z   GAGGTTGGACTTGGGAACACAACACGGCAGTTTTGGTTTGTGACTCCTCCTGAAATCGGTCCCGATGTGCCATATACATTTGGTCTCATAGGTAA 1045
G-GmPAP14N   GAGGTTGGACTTGGGAACACAACACGGCAATTTTGGTTTGTGACTCCTCCTGAAATTGGTCCTGATGTGCCATACACATTTGGTCTCATAGGTAA 1045

G-GmPAP14Z   GTGTACTATTCTATCAAAGTAATTCGGAGTCACTGAGGGGGTCTAGCTCAACTGTCTTTATCACCTTGAATTGTTGAGCATTCATCATTAATTTG 1140
G-GmPAP14N   GTGTACTATTCTATCAAAGTAATTCGGAGTCACTGAGGGGGTCTAGCTCAATTGTCTTTATCACCTTGAATTGTTGAGCATTCATCATTAATTTG 1140

G-GmPAP14Z   GGAAGGAAAAATGCTATATGTTTGTGTACCAATAGGCCATTATGTTTCCTGTAAACTGGTTTGTCTTTATTTGCAGGGGATCTTGGTCAGAGTTT 1235
G-GmPAP14N   GGAAGGAAAAATGCTATATGTTTGTGTACCAATAGGCCATTATGTTTCCTGTAAACTGGTTTGTCTTTATTTGCAGGGGATCTTGGTCAGAGTTT 1235

G-GmPAP14Z   TGATTCAAATAAAACTCTTTCTCACTATGAATTGAACCCAAGAAAAGGACAAACTGTACTATTCGTTGGAGACCTCTCTTATGCGGATAACTACC 1330
G-GmPAP14N   TGATTCAAATAAAACTCTTTCTCACTATGAATTGAACCCAAGAAAAGGACAAACTGTACTATTCGTTGGAGACCTCTCTTATGCGGATAACTACC 1330

G-GmPAP14Z   CAAATCATGATAACATTAGGTGGGATTCTTGGGGAAGGTTTACAGAAAGGAGTGTTGCTTATCAACCATGGATATGGACTGCAGGAAACCATGAA 1425
G-GmPAP14N   CAAATCATGATAACATTAGGTGGGATTCTTGGGGAAGGTTTACAGAAAGGAGTGTTGCTTATCAACCATGGATATGGACTGCAGGAAACCATGAA 1425

G-GmPAP14Z   ATTGATTTTGCTCCAGAAATTGTAAGCTTCTCATAAGAATTGACTTTATTTTGTTTTAACTATCTGAGAAACTCTTCATTCTTTTATTTTTTCAA 1520
G-GmPAP14N   ATTGATTTTGCTCCAGAAATTGTAAGCTTCTCATAAGAATTGACTTTATTTTGTTTTAACTATCTGAGAAACTTTTCATTCTTTTATTTTTTCAA 1520

G-GmPAP14Z   CCATAAATTCTAACCCTCGTGTAATGCATTTTCCTAGTCCAAATTAGTAAGAAGTAAGTTTTTAACATGGATCACTGCTTTGTTGTAGGGTGAAA 1615
G-GmPAP14N   CCATAAATTCTAACCCTCGTGTAATGCATTTTCCTAGTCCAAATTAGTAAGAAGTAAGTTTTTAACATGGATCACTGCTTTGTTGTAGGGTGAAA 1615

G-GmPAP14Z   CTGTACCTTTCAAGCCTTATACCCACCGTTACCATGTTTCTTATAAAGCATCTCAAAGTACTTCACCCTTCTGGTATTCTATCAAGAGAGCTTCA 1710
G-GmPAP14N   CTGTACCTTTCAAGCCTTATACCCACCGTTACCATGTTCCTTATAAAGCATCTCAAAGTACTTCACCCTTCTGGTATTCTATCAAGAGAGCTTCA 1710

G-GmPAP14Z   GCACACATCATTGTTTTGGCCTCATATTCAGCGTATGGTAATGCTAGATGTCTTTTGCATGATACATATTTATGTAGAGAGCACTGGCTGATTCT 1805
G-GmPAP14N   GCACACATCATTGTTTTGGCCTCATATTCAGCGTATGGTAATGCTAGATGTCTTTTGCATGATACATATTTATGTAGAGAGCACTGGCTGATTCT 1805

G-GmPAP14Z   ATGATTTCAGACAAACACGATTCTGTTTGTAATAATTTCACCTTAGTGATTACCTATATGACTTGGAAAAGATCAAATTTTACTGAATAATTTCA 1900
G-GmPAP14N   ATGATTTCAGACAAACACGATTCTGTTTGTAATAATTTCACCTTAGTGATTACCTATATGACTTGGAAAAGATCAAATTTTACTGAATAATTTCA 1900

G-GmPAP14Z   GTTCTTAGATCTATATATATCTATCTGTATGTATGTATGTTTCCATTAAATGTGGAATGTTAA-----ATATCACAGACA--------------- 1975
G-GmPAP14N   GTTCTTAGATCTATATATATCTATCTGTATGTATGTATGTTTCCATTAAATGTGGAATGTTATTGTTTATCTCTAAAGCATGATGTCGGTGTCCT 1995

G-GmPAP14Z   ----------------AACACTTGCCTTAAATGATCTGTGCGTGTGTCTGTGCTAACATGTAAAGTTAAATTGCATTCGTCAGCTGTTCTGTTAC 2054
G-GmPAP14N   TAAGTCCTTGACCAACAACACTTGCCTTAAATGATCTGTGCGTGTGTCTGTGCAAACATGTAAAGTTAAATTGCATTCGTCAGCTGTTCTGTTAC 2090

G-GmPAP14Z   TACTTGGATTAAATGCACACATAATCTGAGATATCATTGTTTTTAAAGTGGTATCTCTTTTGTTACAATAATCTTCCATTTTCTCAGGAAAATAT 2149
G-GmPAP14N   TACTTGGATTAAATGCACACATAATCTGTGATATCATTGTTTTTAAAGTGGTATCTCTTTTGTTACAATAATCTTCCATTTTCTCAGGAAAATAT 2185

G-GmPAP14Z   ACACCACAATATAAATGGCTTGAAAAGGAGCTACCGAAAGTTAACAGGACAGAGACTCCTTGGTTGATTGTTCTCATGCATTCACCTTGGTATAA 2244
G-GmPAP14N   ACACCACAATATAAATGGCTTGAAAAGGAGCTACCGAAAGTTAACAGGACAGAGACTCCTTGGTTGATTGTTCTCATGCATTCACCTTGGTATAA 2280

G-GmPAP14Z   TAGCTACAATTATCACTATATGGAAGGGGAAACAATGAGAGTCATGTATGAGCCCTGGTTTGTGCAGTACAAGGTTGATGTTGTGTTTGCTGGTC 2339
G-GmPAP14N   TAGCTACAATTATCACTATATGGAAGGGGAAACAATGAGAGTCGTGTATGAGCCCTGGTTTGTGCAGTACAAGGTTGATGTTGTGTTCGCTGGTC 2375

G-GmPAP14Z   ATGTTCATGCCTATGAACGATCTGTAAGTGCTAAAACTTGGTCTCTTAAGCTACTAGCACTTCAATTCCTCATTTGAAATTTCTACAACATGTTT 2434
G-GmPAP14N   ATGTTCATGCCTATGAACGATCTGTAAGTGCTAAAACTTGGTCTCTTAAGCTACTAGCACTTCAATTCCTCATTTGAAATTTCTACAACATGTTT 2470

G-GmPAP14Z   TTGAAAGCCGCCGGTATCAATCATGTATCATTAGGCTTGCATTCAAACTTGAAAAGATAACTGTGCAAGTAAATGTCAGTTTCTGGCCCTAAAAA 2529
G-GmPAP14N   CTGAAAGCCGCCGGTATCAATCATGTATCATTAGGCTTGCATTCAAACTTGAAAAGATAACTGTGCAAGTAAATGTCAGTTTCTGGCCCTAAAAA 2565

G-GmPAP14Z   CTTAAGATGAAAATAAGCTACAAAAATATTGCACAAGTAATACACTTCAATAAACTTTTTTGAATTTGTATTTGATTGATGAGCTACTATGTATC 2624
G-GmPAP14N   CTTAAGATGAAAATAAGCTACAAAAATGTTGCACAAGTAATACACTTCAATAAACTTTTTTGAATTTGTATCTGATTGATGAGCTACTATGTATC 2660

G-GmPAP14Z   AGGAGCGTGTTTCCAATGTTGCATACAATATTGTAAATGGTCTTTGTGCTCCTGTAAATGATAAATCAGCTCCTGTATATATAACCATTGGGGAT 2719
G-GmPAP14N   AGGAGCGTGTTTCCAATGTTGCATACAATATTGTAAATGGTCTTTGTGCTCCTGTAAATGATAAATCAGCTCCTGTATATATAACCATTGGGGAT 2755

G-GmPAP14Z   GGAGGAAACCTTGAAGGTTTAGCAACCAAGTAAGTTCATTATATGTAACATGATGAGTCTTGTCACATTTTTTTTTATTTTTATGCCTCTAAGTT 2814
G-GmPAP14N   GGAGGAAACCTTGAAGGTTTAGCAACCAAGTAAGTTCATTATATGTAACATGATGAGTCTTGTCATATTTCTTCTTATTTTTATGCCTCTAAGTC 2850

G-GmPAP14Z   TGATATAAATGACATGAGTTGCAGCATGACAGAACCACAGCCAAAGTATTTTACATTCCGAGAGGCCAGCTTTGGACATGCCATTTTTGACATAA 2909
G-GmPAP14N   TGATATAAATGACATGAGTTGCAGCATGACAGAACCACAGCCAAAGTATTCTGCATTCCGAGAGGCCAGCTTTGGACATGCCATTTTTGACATAA 2945

G-GmPAP14Z   CGAACCGAACTCATGCTCACTATAGCTGGCACCGAAATCAAGATGGAGTTGCTGTGGAGGCCGATTCCCTTTGGTTTTTCAACAGATACTGGCAC 3004
G-GmPAP14N   CGAACCGAACTCATGCTCACTATAGCTGGCACCGAAATCAAGATGGAGTTGCTGTGGAGGCCGATTCCCTTTGGTTTTTCAACAGATACTGGCAC 3040

G-GmPAP14Z   CCAGTTGATGATTCCACGGCTCATGTTTCACATTAA 3040
G-GmPAP14N   CCAGTTGATGATTCCACGGCTCATGTTTCACATTAA 3076
```

Figure S2 : Sequence alignment of *G-GmPAP14Z* and *G-GmPAP14N*.

```
GmPAP14Z ATGGGTGTTGTGGAGGGTCTCTTAGCATTGGCTTTGGTTCTGAGTGTTTGCGTGGTGTGCAATGGAGGCTCAAGCAGCCC 80
GmPAP14N ATGGGTGTTGTGGAGGGTCTCTTAGCATTGGCTTTGGTTCTGAGTGTTTGCGTGGTGTGCAATGGAGGCTCAAGCAGCCC 80

GmPAP14Z CTTCATTAGGAAAGTTGAGAAGACAGTGGATATGCCACTTGACAGCGATGTCTTTGCTGTTCCTCCTGGTTATAATGCTC 160
GmPAP14N CTTCATTAGGAAAGTTGAGAAGACAGTGGATATGCCACTTGACAGCGATGTCTTTGCTGTTCCTCCTGGTTATAATGCTC 160

GmPAP14Z CCCAGCAGGTTCATATAACGCAAGGTGACCTTGTGGGGAAAGCAGTGATCGTGTCATGGGTGACAGTGGATGAACCGGGG 240
GmPAP14N CCCAGCAGGTTCATATAACGCAAGGTGACCTTGTGGGGAAAGCAGTGATCGTGTCATGGGTGACAGTGGATGAACCGGGG 240

GmPAP14Z TCGAGCGAAGTGCATTACTGGAGTGAGAACAGTGACAAGAAGAAGATTGCCGAAGGAAAACTTGTTACTTATAGGTTCTT 320
GmPAP14N TCGAGCGAAGTGCATTACTGGAGTGAGAACAGTGACAAGAAGAAGATTGCCGAAGGAAAACTTGTTACTTATAGGTTCTT 320

GmPAP14Z CAATTACTCATCTGGGTTTATACACCACACCACCATCAGAAATTTGGAGTACAAAACCAAATACTACTATGAGGTTGGAC 400
GmPAP14N CAATTACTCATCTGGGTTTATACACCACACCACCATCAGAAATTTGGAGTACAAAACCAAATACTACTATGAGGTTGGAC 400

GmPAP14Z TTGGGAACACAACACGGCAATTTTGGTTTGTGACTCCTCCTGAAATTGGTCCTGATGTGCCATACACATTTGGTCTCATA 480
GmPAP14N TTGGGAACACAACACGGCAATTTTGGTTTGTGACTCCTCCTGAAATTGGTCCTGATGTGCCATACACATTTGGTCTCATA 480

GmPAP14Z GGGGATCTTGGTCAGAGTTTTGATTCAAATAAGACTCTTTCTCACTATGAATTGAACCCAAGAAAAGGACAAACTGTACT 560
GmPAP14N GGGGATCTTGGTCAGAGTTTTGATTCAAATAAAACTCTTTCTCACTATGAATTGAACCCAAGAAAAGGACAAACTGTACT 560

GmPAP14Z GTTTGTTGGAGACCTCTCTTATGCGGATAACTACCCAAATCATGATAACGTTAGGTGGGATTCTTGGGGAAGGTTTACAG 640
GmPAP14N ATTCGTTGGAGACCTCTCTTATGCGGATAACTACCCAAATCATGATAACATTAGGTGGGATTCTTGGGGAAGGTTTACAG 640

GmPAP14Z AAAGGAGTGTTGCTTATCAACCATGGATATGGACTGCAGGAAACCATGAAATTGATTTTGCCCCAGAAATTGGTGAAACC 720
GmPAP14N AAAGGAGTGTTGCTTATCAACCATGGATATGGACTGCAGGAAACCATGAAATTGATTTTGCTCCAGAAATTGGTGAAACT 720

GmPAP14Z GTACCTTTCAAACCTTATACCCACCGTTACCATGTTCCTTATAAAGCATCTCAAAGTACTTCACCCTTCTGGTATTCTAT 800
GmPAP14N GTACCTTTCAAGCCTTATACCCACCGTTACCATGTTCCTTATAAAGCATCTCAAAGTACTTCACCCTTCTGGTATTCTAT 800

GmPAP14Z CAAGAGAGCTTCAGCACACATCATTGTTTTGGCCTCATATTCAGCATATGGAAAATATACACCACAATATAAATGGCTTG 880
GmPAP14N CAAGAGAGCTTCAGCACACATCATTGTTTTGGCCTCATATTCAGCGTATGGAAAATATACACCACAATATAAATGGCTTG 880

GmPAP14Z AAGAGGAGCTACCGAAAGTTAACAGGACAGAGACTCCTTGGTTGATTGTTCTGATGCATTCACCTTGGTATAATAGCTAC 960
GmPAP14N AAAAGGAGCTACCGAAAGTTAACAGGACAGAGACTCCTTGGTTGATTGTTCTCATGCATTCACCTTGGTATAATAGCTAC 960

GmPAP14Z AATTATCACTATATGGAAGGGGAAACAATGAGAGTAATGTATGAGCCCTGGTTTGTGCAGTACAAGGTTGATGTTGTTTT 1040
GmPAP14N AATTATCACTATATGGAAGGGGAAACAATGAGAGTCGTGTATGAGCCCTGGTTTGTGCAGTACAAGGTTGATGTTGTGTT 1040

GmPAP14Z TGCTGGTCATGTTCATGCCTATGAACGATCTGAGCGTGTTTCCAATGTTGCATACAATATTGTAAATGGTCTTTGTGCTC 1120
GmPAP14N CGCTGGTCATGTTCATGCCTATGAACGATCTGAGCGTGTTTCCAATGTTGCATACAATATTGTAAATGGTCTTTGTGCTC 1120

GmPAP14Z CTGTAAAAGATCAATCAGCTCCTGTATATATAACCATTGGTGATGGAGGAAACCTTGAAGGTTTAGCAACCAACATGACA 1200
GmPAP14N CTGTAAATGATAAATCAGCTCCTGTATATATAACCATTGGGGATGGAGGAAACCTTGAAGGTTTAGCAACCAACATGACA 1200

GmPAP14Z GAACCACAGCCAGAGTATTCTTCATTCCGAGAGGCCAGCTTTGGACATGCCATTTTTGACATAACGAACCGAACTCATGC 1280
GmPAP14N GAACCACAGCCAAAGTATTCTGCATTCCGAGAGGCCAGCTTTGGACATGCCATTTTTGACATAACGAACCGAACTCATGC 1280

GmPAP14Z TCACTACAGCTGGCACCGAAATCAAGACGGAGTTGCTTTCGAGGCTGATTCCGTTTGGTTTTTCAACAGATACTGGCACC 1360
GmPAP14N TCACTATAGCTGGCACCGAAATCAAGATGGAGTTGCTGTGGAGGCCGATTCCCTTTGGTTTTTCAACAGATACTGGCACC 1360

GmPAP14Z CAGTTGATGATTCCACGGCTCATGTTTCACATTAA                                              1395
GmPAP14N CAGTTGATGATTCCACGGCTCATGTTTCACATTAA                                              1395
```

Figure S3 : Sequence alignment of *GmPAP14Z* and *GmPAP14N*.
